# Case report: A persistently expanded T cell response in an exceptional responder to radiation and atezolizumab for metastatic non-small cell lung cancer

**DOI:** 10.3389/fimmu.2022.961105

**Published:** 2022-09-09

**Authors:** David G. Coffey, Yuexin Xu, Andrea M. H. Towlerton, Marcin Kowanetz, Priti Hegde, Martine Darwish, Mahesh Yadav, Craig Blanchette, Shannon M. Ruppert, Sarah Bertino, Qikai Xu, Andrew Ferretti, Adam Weinheimer, Matthew Hellmann, Angel Qin, Dafydd Thomas, Edus H. Warren, Nithya Ramnath

**Affiliations:** ^1^ Department of Medicine, Fred Hutchinson Cancer Center, Seattle, WA, United States; ^2^ Department of Medicine, Sylvester Comprehensive Cancer Center, Miami, FL, United States; ^3^ ArriVent Biopharma, Newtown Square, PA, United States; ^4^ Foundation Medicine, Cambridge, MA, United States; ^5^ Genentech, San Francisco, CA, United States; ^6^ TScan Therapeutics, Waltham, MA, United States; ^7^ AstraZeneca, Wilmington, DE, United States; ^8^ Department of Medicine, University of Michigan, Ann Arbor, MI, United States; ^9^ Department of Pathology, University of Michigan, Ann Arbor, MI, United States; ^10^ Precision Oncology Program, Veterans Affairs, Ann Arbor Healthcare System, Ann Arbor, MI, United States

**Keywords:** case report, immunotherapy, programmed death-ligand 1, t lymphocytes, lung neoplasms

## Abstract

Most patients with advanced non-small cell lung cancer (NSCLC) do not achieve a durable remission after treatment with immune checkpoint inhibitors. Here we report the clinical history of an exceptional responder to radiation and anti-program death-ligand 1 (PD-L1) monoclonal antibody, atezolizumab, for metastatic NSCLC who remains in a complete remission more than 8 years after treatment. Sequencing of the patient’s T cell repertoire from a metastatic lesion and the blood before and after anti-PD-L1 treatment revealed oligoclonal T cell expansion. Characterization of the dominant T cell clone, which comprised 10% of all clones and increased 10-fold in the blood post-treatment, revealed an activated CD8^+^ phenotype and reactivity against 4 HLA-A2 restricted neopeptides but not viral or wild-type human peptides, suggesting tumor reactivity. We hypothesize that the patient’s exceptional response to anti-PD-L1 therapy may have been achieved by increased tumor immunogenicity promoted by pre-treatment radiation therapy as well as long-term persistence of oligoclonal expanded circulating T cells.

## Introduction

Immunotherapy using immune checkpoint inhibitors has revolutionized treatment of various solid organ cancers, including non-small cell lung cancer (NSCLC). These therapies include monoclonal antibodies targeting immune checkpoint receptors anti-program death-1 (PD-1) and cytotoxic T-lymphocyte-associated protein 4 (CTLA-4) as well as tumor ligand anti-program death-ligand 1(PD-L1) (1). Durable clinical benefit with response lasting greater than 6 months is described in 20% of patients with stage IV NSCLC (2–5). Notably, 5-year survival of 15% has been reported with anti-PD1 based therapies for metastatic NSCLC (6, 7). Few studies have described long term persistence of expanded T-cell clonotypes in these long-term survivors. The current study provides a comprehensive evaluation of the T cell receptor β (TCRβ) repertoire and characterization of an expanded T cell clone in an exceptional responder to radiation and anti-PD-L1 antibody, atezolizumab. The patient is currently alive with no evidence of cancer 11 years following diagnosis of metastatic NSCLC.

## Case description

A 63-year-old, asymptomatic woman with prior stage I, estrogen receptor positive, invasive ductal carcinoma of the left breast was found to have a right lower lobe lung nodule and enlarged right hilar lymph node upon imaging for breast cancer surveillance. She reported a prior history of smoking but had no family history of cancer. Full body imaging uncovered numerous bony lesions and a solitary right parietal lobe mass within the brain. Biopsy of the left iliac bone confirmed TTF-1 positive malignant cells consistent with adenocarcinoma of the lung. Targeted DNA sequencing of the tumor (FoundationOne) identified alterations in 23 of 236 assayed genes, including *KRAS* G12F and *BRAC2* P1819S. No mutations were identified in *EGFR*. She was treated with stereotactic radiosurgery to the brain lesion followed by carboplatin, pemetrexed and bevacizumab chemotherapy. A year later, she underwent stereotactic body radiation therapy (SBRT) to a residual right lower lobe lung mass and achieved a near complete response.

**Figure 1:**
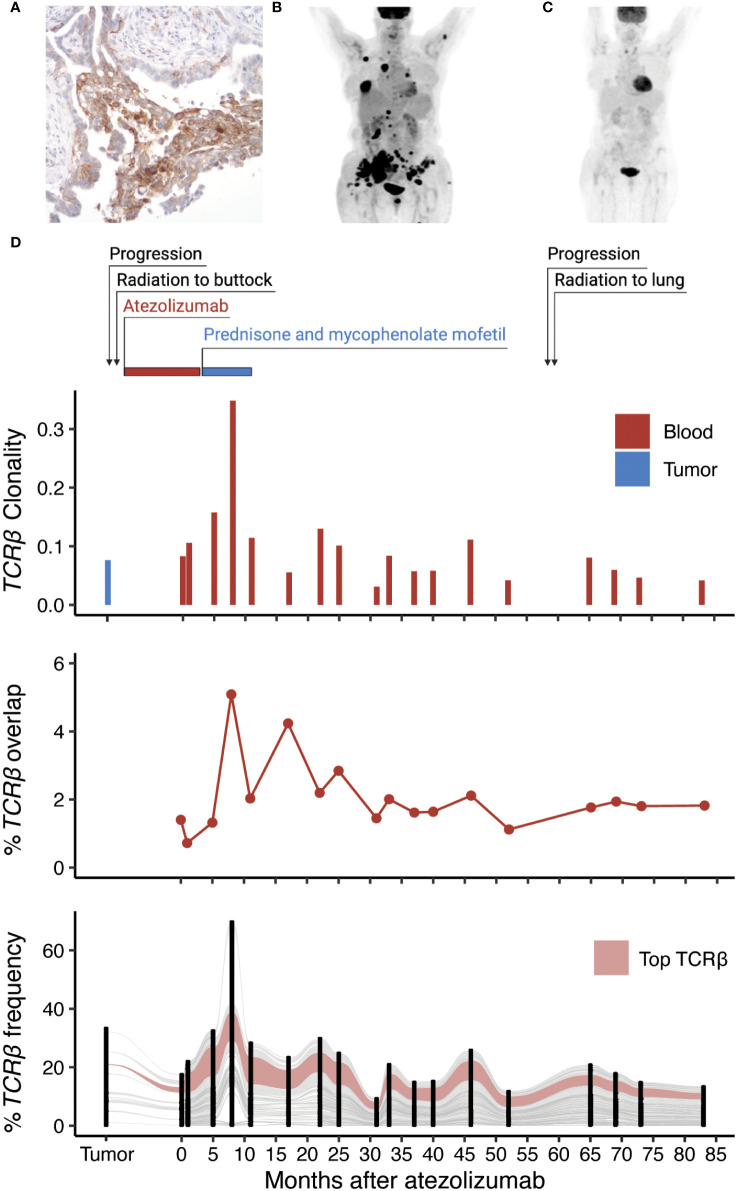
Identification of an expanded T cell clone following radiation and anti-PD-L1 therapy. **(A)** Expression of PD-L1 within the metastatic tumor biopsy by immunohistochemistry. **(B)** PET scan of the patient before and **(C)** one year of anti-PD-L1 therapy. **(D)** Treatment timeline of the patient in relation to TCRβ clonality (inverted Shannon entropy), percent overlap of all TCRβ sequences detected in the tumor and the blood, and an alluvial plot tracking the frequency of the top 100 TCRβ sequences shared by at least 2 samples across all collection time points.

Eight months later, she was found to have increasing lung nodules around the site of radiation. A positron emission tomography (PET) scan revealed increased uptake in the right lower lobe of the lung, right hilum, right axilla and numerous bony sites. Despite treatment with pemetrexed, she continued to progress and developed vaginal wall lesions and a new subcutaneous lesion within the right buttock. Biopsy of this lesion confirmed lung adenocarcinoma. She received palliative radiation to the right buttock and enrolled in a single-arm, phase II study evaluating the efficacy and safety of atezolizumab, an anti-PD-L1 inhibitor, in patients with locally advanced or metastatic NSCLC (NCT01846416) (8). Immunohistochemistry of her right buttock tumor biopsy confirmed 100% of her cancer cells expressed PD-L1 (C223 DAKO monoclonal antibody, **Figure 1A**). Repeat imaging prior to her enrollment in the clinical trial revealed further progression of her cancer with nodal and lymphangitic spread (**Figure 1B**).

After the third dose of atezolizumab, she developed symptoms of hypothyroidism that was treated thyroid replacement hormone. Subsequently, she noted hypopigmentation in the region of her right buttock, where she had previously been irradiated. She also developed skin tightening along her torso, forearms, antecubital fossa, and posterior legs. A skin biopsy of the left forearm showed slight collagen alteration and an eosinophilic infiltrate. She was found to have peripheral eosinophilia with absolute eosinophil count of 1.4 cells/µL (normal less than 0.5 cells/µL). She was diagnosed with eosinophilic fasciitis and recommended to discontinue atezolizumab since it was believed to be the cause of her skin reaction. She was treated with prednisone resulting in substantial improvement in the appearance and thickening of her skin and then transitioned to mycophenolate mofetil. Following her last dose of atezolizumab, PET imaging revealed a complete resolution of her cancer including the lymph nodes, primary tumor, and bone metastases (**Figure 1C**). Four years later, an enlarging right upper lobe lesion within the lung was identified and treated with empiric SBRT since the site was inaccessible for a biopsy. Over the subsequent three years the patient has remained in a complete remission with no detectable disease on annual radiographic imaging.

## Characterization of the T cell repertoire and dominant T cell clone

To characterize the T cell response of this exceptional responder, we performed sequencing of the TCRβ chain on genomic DNA extracted from the patient’s metastatic subcutaneous tumor biopsy in addition to 18 peripheral blood samples including one obtained before and 12 months after treatment with atezolizumab (**Supplemental Methods**). Additionally, TCRβ sequencing was performed on sorted CD3^+^CD4^+^ and CD3^+^CD8^+^ T cells from one sample obtained 19 months after immunotherapy. Pre-treatment metastatic skin lesions showed greater TCRβ diversity than the post-treatment blood samples (**Figure 1D**). Among the 2,163 productive TCRβ sequences detected in the pre-treatment metastatic skin lesions, 52.5% were also detected in at least one of the post-treatment blood samples. Most T cell clones present in the tumor increased in frequency from the pre-treatment to the post-treatment blood samples and were also detected in both the CD4^+^ and CD8^+^ sorted blood samples suggesting a polyclonal immune response. The most abundant peripheral blood complementarity-determining region 3 β (CDR3β) sequence detected (CASSLERGLAVSGANVLTF) increased 10-fold in frequency in the 12 months after anti-PD-L1 therapy and was present within the sorted CD8^+^ T cell population, but not the CD4^+^ T cell population. The frequency of this sequence among productive CDR3β sequences was 0.24% in the pre-treatment skin biopsy, 1.01% in the pre-treatment blood sample and increased to 10.5% 12 months after treatment (42 months post diagnosis). We continued to observe this sequence in every blood sample at relatively stable frequency, including the last sample 83 months after treatment (113 months post diagnosis). Sequencing of the TCRβ repertoire from the patient’s biopsy revealing eosinophilic fasciitis did not detect the dominant clone. Single-cell RNA sequencing (scRNAseq) of CD3+ sorted blood cells confirmed the dominant CDR3β sequence paired with the CDR3α sequence CIVGVHYGGSQGNLIF.

**Figure 2:**
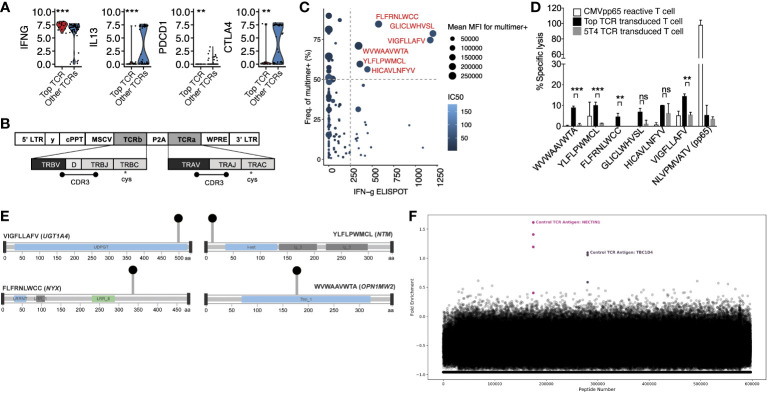
Phenotypic characterization and antigen specificity of the dominant T cell clone. **(A)** Differential gene expression from targeted scRNAseq comparing the dominant (“Top TCR”) to non-dominant clones. Significance was measured by T test. **(B)** Construction of TCRαβ clone. **(C)** Interferon-gamma elispot in relation to mean fluorescence intensity (MFI) of neopeptide generated tetramers. Dashed lines define threshold for reactive peptides (show in red) **(D)** Chromium release assay of candidate neopeptides. Mean with standard deviations are plotted along with the level of significance from T tests. **(E)** Lollipop plots showing the location of non-synonymous mutations within the protein amino acid sequence of candidate neoantigens. **(F)** T-Scan results showing the fold enrichment of each protein fragment in the T-Scan peptidome library. Each point represents an individual protein fragment. Enriched protein fragments with a common epitope are highlighted and labeled. ns: P > 0.05, *P ≤ 0.05, **P ≤ 0.01, ***P ≤ 0.001.

Having identified the α and β CDR3 sequences of the dominant T cell clone that was present in the tumor and expanded in the blood after anti-PD-L1 therapy, we next characterized its phenotype and identified its antigenic specificity. Targeted single-cell RNA sequencing using a 23 gene panel, confirmed the T cell to be CD8^+^ and we identified increased differential expression of *IFNG* and reduced expression of *IL13*, *PDCD1*, and *CTLA4* (**Figure 2A**) compared with other T cells derived from the tumor. Whole-exome sequencing of the tumor for neoantigen prediction identified 792 unique neopeptides encoded by 201 genes. We cloned the dominant αβ TCR (**Figure 2B**) and transduced it into donor CD8^+^ human T cells and screened it for reactivity against the top 165 HLA-A2 restricted neopeptides. Additionally, we constructed HLA-A2 specific tetramers using these 165 neopeptides by UV mediated peptide exchange (9) and screened for affinity to the dominant clone (**Figure 2C**). This combined analysis identified 6 candidate neopeptides which we validated using a chromium release assay (**Figure 2D**). This analysis revealed 4 statistically significant neopeptides with reactivity to the patient’s dominant T cell clone. These neopeptides are encoded by the genes *NTM*, *UGT1A4*, *OPN1MW2*, and *NYX* (**Figure 2E**). Finally, to exclude the possibility that the dominant T cell clone also reacts to a self-antigen, we performed a T-Scan analysis against the entire viral and human peptidome. No protein fragments were detected besides known fragments recognized by the included positive control TCR suggesting the dominant T cell clone does not recognize a self-antigen (**Figure 2F**) (10). Taken together, these results suggest that the expanded T cell clone was an activated, effector CD8^+^ T cell with reactivity against tumor-derived neopeptides.

## Discussion

We present a case of a patient with widely metastatic *EGFR* wild-type lung adenocarcinoma who achieved a durable complete response following radiation and anti-PD-L1 therapy. The mutation of a DNA repair gene within her tumor cells may have increased the mutational burden within her tumor, resulting in a higher neoantigen load. Additionally, we hypothesize that the radiation therapy administered prior to atezolizumab may have acted synergistically with the immunotherapy to augment her response. Prior studies have shown that radiation therapy can enhance tumor immunogenicity, overcome the immunosuppressive effects of the tumor microenvironment, and increase recruitment of antigen-presenting and immune effector cells to the tumor microenvironment (11). Additionally, multiple clinical studies have demonstrated improved treatment outcomes among patients with lung cancer receiving radiation combined with immune checkpoint inhibitors compared to immune checkpoint inhibitors alone (12–14). Serial TCRβ sequencing of the blood revealed polyclonal expansion of CD8^+^ and CD4^+^ T cell clones within the pre-treatment metastatic tumor biopsy including a single CD8^+^ T cell clone that expanded more than 10-fold. Single-cell and antigen characterization of the dominant T cell clone demonstrated the T cell was likely tumor specific based on its activated, *IFNG* expressing CD8^+^ phenotype and neopeptide reactivity.

Development of autoimmune vitiligo after immunotherapy in our patient may have been an early indicator of effective treatment response. While vitiligo is not a commonly reported adverse effect of checkpoint inhibitor therapy in lung cancer (15, 16), it is a relatively common event in melanoma (17) and associated with improved treatment outcome (18, 19). Resident memory T cells (T_RM_) specific to melanoma antigens have been reported to be maintained in vitiligo-affected skin of melanoma patients (20). One study demonstrated long-term persistence of T_RM_ cell clones in the skin and effector memory T (T_EM_) cells in the blood among vitiligo-affected patients with melanoma and exceptional responses to immunotherapy (21). T_RM_ are also associated with long-lived protection against infection (22) as wells inflammatory conditions of the skin (23). These observations may suggest that the expanded T cell clones we detected in the patient’s metastatic skin biopsy may have had a memory phenotype that contributed to their long term persistence. Although we did not detect reactivity of the patients dominate T cell clone to viral peptides screened by the T-Scan assay, it is possible some of the tumor infiltrating T cell clones may have cross reactivity to microbial antigens which would explain their long-term persistence. Others have reported that virus-specific memory T cells can extend their surveillance to neoantigens expressed by the tumor cells (24–26).

The observation that the patient’s dominant T cell clone reacted to multiple neopeptide-MHC complexes is not surprising. In fact, it is believed that a single TCR can recognize >10^6^ different MHC-associated epitopes (27). Its ability to cross-react with multiple tumor antigens may have increased its probability of reacting to the tumor, especially if the mutations encoding the neopeptides were not present in all tumor subclones. Perhaps it is for this reason that the T cell exhibited the highest degree of expansion, enabling it to become the dominant clone.

## Data availability statement

The datasets presented in this study can be found in online repositories. The names of the repository/repositories and accession number(s) can be found in the article/**Supplementary Material**.

## Ethics statement

This study was reviewed and approved by University of Michigan Institutional Review Board. The patients/participants provided their written informed consent to participate in this study. Written informed consent was obtained from the individual(s) for the publication of any potentially identifiable images or data included in this article.

## Author contributions

Study conception and design: DC, YX, EW, NR. Data collection: DC, YX, AT, MK, MD, MY, CB, SR, SB, QX, AF, AW, MH, DT. Analysis and interpretation of results: DC, YX, AT, MK, MD, MY, CB, SR, SB, QX, AF, AW, MH, EW, NR. Draft manuscript preparation: DC, YX, AT, CB, AF, AQ, EW, NR. All authors contributed to the article and approved the submitted version.

## Funding

This research was supported by funding from the Cancer Therapeutics Endowment. This research was also supported by the Flow Cytometry and Genomics Shared Resources of the Fred Hutch/University of Washington Cancer Consortium (P30 CA015704). Bioinformatic analysis was supported by the Scientific Computing Infrastructure at Fred Hutch funded by ORIP grant S10OD028685.

## Conflict of interest

MK was employed by ArriVent. PH was employed by Foundation Medicine. MD, MY, CB, and SB were employed by Genetech. QX, AF, AW were employed by Tscan.

The remaining authors declare that the research was conducted in the absence of any commercial or financial relationships that could be construed as a potential conflict of interest.

## Publisher’s note

All claims expressed in this article are solely those of the authors and do not necessarily represent those of their affiliated organizations, or those of the publisher, the editors and the reviewers. Any product that may be evaluated in this article, or claim that may be made by its manufacturer, is not guaranteed or endorsed by the publisher.
