# Supplementary material for: Case report: A persistently expanded T cell response in an exceptional responder to radiation and atezolizumab for metastatic non-small cell lung cancer
Source: Front Immunol. 2022 Sep 9;13:961105. doi: 10.3389/fimmu.2022.961105 (PMC9500393; doi:10.3389/fimmu.2022.961105)
Supplement: Supplementary file 1 [file DataSheet_1.pdf]

Case Reports: A Persistently Expanded T Cell Response in an Exceptional Responder to Radiation and Atezolizumab for Metastatic Non-Small Cell Lung Cancer

SUPPLEMENTAL METHODS

Identification and cloning of the dominant T cell clone

Peripheral blood mononuclear cells (PBMC) were isolated from blood by ficoll density gradient centrifugation. DNA was extracted from blood cells using the QIAmp Blood Mini Kit (QIAGEN) and from formalin-fixed paraffin-embedded (FFPE) tumor samples using the QIAamp DNA FFPE Tissue Kit (QIAGEN). $CD3^+CD8^+$ and $CD3^+CD4^+$ T cells were sorted by fluorescence-activated cell sorting (FACS) using an ARIAII (BD Biosciences). Next-generation sequencing of the CDR3β chain of the T cell receptor was performed using the ImmunoSEQ hsTCRB v3.0 assay (Adaptive Biotechnologies). T cell repertoire analysis was performed using LymphoSeq(1) and data have been made available online (https://clients.adaptivebiotech.com/immuneaccess). Targeted scRNAseq of individual flow sorted T cells was performed as previously described.(2) Briefly, $CD3^+$ T cells were sorted into a 96-well plate, lysed, and cDNA was synthesized. Nested PCR was performed using a panel of primers targeting 23 phenotypic genes and all variable genes with the *TRB* and *TRA* loci (supplemental table 1). Libraries were sequenced on a MiSeq (Illumina) to generate 150 bp, paired-end reads. The dominant αβ CDR3 sequences were synthesized by GeneArt Gene Synthesis (Invitrogen) and cloned into a PPLSIN vector driven by a MSCV promotor (a generous gift from Philip Greenberg, Fred Hutchinson Cancer Center). Lentiviral packaging and human $CD8^+$ T cell transduction were performed.

Neoantigen prediction

Whole exome sequencing of the patient's metastatic tumor biopsy was performed and non-synonymous somatic variants were called. Neopeptide prediction was performed from 9-mer amino acid sequences generating using a sliding window approach such that each mutation was contained at every position within the peptide (supplemental table 2). HLA typing was performed by next-generation sequencing using the ScisGo HLA kit (Sisco Genetics). Since the patient was found to express HLA-A*02:01, the binding affinity of each 9-mer neopeptide to HLA-A*02:01 was predicted using NetMHCpan 4.1. The peptides were ranked according to the HLA-A*02:01 binding affinity and mean TCGA mRNA gene expression. The top 165 neopeptides with the highest binding affinity (lowest IC50) and greatest TCGA expression were synthesized.

Evaluation of dominant T cell clone antigen specificity

Interferon-gamma ELISA were performed on the supernatant of pulled peptides pulsed with T2 cells and transgenic TCR transduced human $CD8^+$ T cells after 16 hours of co-culture using the human Interferon-Gamma PicoKine ELISA kit (Boster Bio). MHC-peptide tetramers were generated from biotinylated MHC UV cleavable monomers by peptide exchange.(3) TCR transduced human $CD8^+$ T cells were tetramer stained and analyzed by FACS. Chromium release assays were performed on the double positive hits identified by the ELISA interferon-gamma and tetramer staining. T-Scan was used to screen for reactivity to normal human proteins.(4) TCR transduced human $CD8^+$ T cells were co-cultured with a library of target cells engineered to express HLA-A*02:01 and a single member of an overlapping protein fragment library. The protein fragment

library spans the entire annotated human proteome in overlapping 90 amino acid fragments. Early apoptotic cells were identified using a granzyme activated reporter and sorted by FACS. The sorted cells were sequenced, and fold enrichment of each fragment was calculated as the fold change of the frequency of the fragment in the sorted sample over the frequency of the fragment in the input library.

REFERENCES

1. Kanakry CG, Coffey DG, Towlerton AMH, Vulic A, Storer BE, Chou J, Yeung CCS, Gocke CD, Robins HS, O'Donnell PV, et al. Origin and evolution of the T cell repertoire after posttransplantation cyclophosphamide. *JCI Insight* (2016) 1: doi: 10.1172/jci.insight.86252

2. Han A, Glanville J, Hansmann L, Davis MM. Linking T-cell receptor sequence to functional phenotype at the single-cell level. *Nature Biotechnology* (2014) 32:684–692. doi: 10.1038/nbt.2938

3. Darwish M, Wichner S, Li J, Chang JC, Tam C, Franke Y, Li H, Chan P, Blanchette C. High-throughput identification of conditional MHCI ligands and scaled-up production of conditional MHCI complexes. *Protein Sci* (2021) 30:1169–1183. doi: 10.1002/pro.4082

4. Kula T, Dezfulian MH, Wang CI, Abdelfattah NS, Hartman ZC, Wucherpfennig KW, Lyerly HK, Elledge SJ. T-Scan: A Genome-wide Method for the Systematic Discovery of T Cell Epitopes. *Cell* (2019) 178:1016-1028.e13. doi: 10.1016/j.cell.2019.07.009
